# Follicle‐stimulating hormone in postmenopausal women living with HIV: a prevalence study

**DOI:** 10.1111/hiv.13205

**Published:** 2021-11-17

**Authors:** Shema Tariq, Hajra Okhai, Abigail Severn, Caroline A. Sabin, Fiona Burns, Richard Gilson, Julie Fox, Yvonne Gilleece, Nicola E. Mackie, Frank A. Post, Iain Reeves, Melanie Rosenvinge, Ann Sullivan, Andrew Ustianowski, Robert F. Miller

**Affiliations:** ^1^ UCL Institute for Global Health London UK; ^2^ Mortimer Market Centre CNWL NHS Foundation Trust London UK; ^3^ National Institute for Health Research (NIHR) Health Protection Research Unit (HPRU) in Blood Borne and Sexually Transmitted Infections at UCL London UK; ^4^ Royal Free London NHS Foundation Trust London UK; ^5^ Guys and St Thomas NHS Foundation Trust London UK; ^6^ University Hospitals Sussex NHS Trust Brighton UK; ^7^ Brighton & Sussex Medical School Brighton UK; ^8^ Imperial College Healthcare NHS Trust London UK; ^9^ Kings College Hospital NHS Foundation Trust London UK; ^10^ Homerton University Hospital NHS Foundation Trust London UK; ^11^ Lewisham and Greenwich NHS Trust London UK; ^12^ Chelsea and Westminster NHS Foundation Trust London UK; ^13^ North Manchester General Hospital Manchester University NHS Foundation Trust Manchester UK

**Keywords:** ageing, follicle‐stimulating hormone, HIV, menopause, women

## Abstract

**Objectives:**

We examined follicle‐stimulating hormone (FSH) levels in women living with HIV aged > 45 reporting ≥ 12 months’ amenorrhoea, and investigated correlation with menopausal symptoms.

**Methods:**

A cross‐sectional substudy of 85 women from the Positive Transitions through the Menopause (PRIME) Study who reported irregular periods at entry into the PRIME Study and ≥ 12 months’ amenorrhoea at recruitment into this substudy. Serum FSH was supplemented with clinical data and menopausal symptom assessment. Serum FSH > 30 mIU/mL was defined as consistent with postmenopausal status. Associations between FSH and menopausal symptom severity were assessed using Pearson's correlation and the Kruskal–Wallis test.

**Results:**

Median age was 53 years [interquartile range (IQR): 51–55]; all were on antiretroviral therapy, three‐quarters (*n* = 65) had a CD4 T‐cell count > 500 cells/μL and 91.8% (*n* = 78) had an HIV viral load (VL) < 50 copies/mL. Median FSH was 65.9 mIU/mL (IQR: 49.1–78.6). Only four women (4.7%) had FSH ≤ 30 mIU/mL; none reported smoking or drug use, all had CD4 T‐cell count ≥ 200 cells/μL, and one had viral load (VL) ≥ 50 copies/mL. Median body mass index (BMI) was elevated compared with women with FSH > 30 mIU/mL (40.8 vs. 30.5 kg/m^2^).

Over a quarter (28.2%) reported severe menopausal symptoms, with no correlation between FSH and severity of menopausal symptoms (*p* = 0.21), or hot flushes (*p* = 0.37).

**Conclusions:**

Four women in this small substudy had low FSH despite being amenorrhoeic; all had BMI ≥ 35 kg/m^2^. We found that 95% of women with HIV aged > 45 years reporting ≥ 12 months’ amenorrhoea had elevated FSH, suggesting that menopausal status can be ascertained from menstrual history alone in this group.

## INTRODUCTION

Improvements in survival due to antiretroviral therapy (ART) have resulted in successfully treated individuals with HIV now having a near normal life expectancy [1, 2], with a consequent shift in the age distribution of people living with HIV. The transformation of HIV into a long‐term, manageable medical condition means that comorbid conditions and other age‐related events are increasingly important; among women, this includes menopause. By 2018, 11 100 women of potentially menopausal age (45–56 years) were attending for HIV care in the UK, a five‐fold increase from 2008 (J Khawam, personal communication, 01 October 2021).

Menopause is a normal life transition but one that can be associated with a range of symptoms and longer‐term health consequences. National Institute for Health and Care Excellence (NICE) guidelines advise that, in women aged > 45 years, menopause is a clinical diagnosis, established after ≥ 12 months’ amenorrhoea in those with an intact uterus and not using hormonal contraception [3]. Biomarkers of ovarian function include anti‐Müllerian hormone (AMH) and follicle‐stimulating hormone (FSH). In the early stages of menopause, FSH levels rise in response to the depletion of ovarian follicles and the consequent fall in levels of Inhibin B [4]. Mean FSH levels increase significantly across the menopause transition, from 7.0 mIU/mL in the reproductive stage to 45.7 mIU/mL in postmenopausal women, meaning that FSH can provide useful biological confirmation of postmenopausal status [5]. However, given the likelihood of menopause being the cause of secondary amenorrhoea in women aged > 45 years, and the fluctuation in FSH levels, laboratory testing for biological markers of ovarian activity is not routinely recommended in amenorrhoeic women in this age group.

It is unclear whether NICE recommendations that menopause be determined by menstrual history alone (without biological confirmation) can be applied to women living with HIV. Prolonged amenorrhoea and anovulatory cycles for reasons other than menopause are common in women living with HIV [6, 7, 8, 9, 10, 11, 12, 13]. A recent meta‐analysis estimated the prevalence of secondary amenorrhoea in women living with HIV to be 4.8%, finding a significant association between HIV status and amenorrhoea [12]. However, it is important to note that the median age of participants in the six studies included was 33–37 years [12]. Recreational drug use, psychotherapeutic medication, lower CD4 T‐cell count, low body mass index (BMI), hepatitis B coinfection, current smoking and taking antiretroviral therapy (ART) have all been shown to be associated with amenorrhoea in this patient population [6, 8, 9, 12, 13].

It is important to be able to assess menopausal status accurately in women living with HIV in order to provide guidance around symptom control (including the appropriate offering of hormone replacement therapy) and prevention of comorbidities (including assessment of low bone mineral density and cardiovascular risk). We require evidence to support the diagnosis of menopause by menstrual pattern alone in women living with HIV aged > 45 years. Furthermore, given the overlap between menopausal and HIV‐related symptoms (such as fatigue, mood changes, pain and night sweats), it is important to understand if a biomarker such as FSH has a role in ascertaining aetiology of symptoms. The aim of this study was to examine FSH levels in women living with HIV aged > 45 years reporting ≥ 12 months’ amenorrhoea, and to investigate correlations with menopausal symptom severity, and hot flush severity specifically.

## METHODS

Between January and December 2019 we conducted a cross‐sectional study of serum FSH in a subsample of women from the Positive Transitions through the Menopause (PRIME) Study [14], a mixed‐methods observational study of the menopause in women living with HIV in England. The main PRIME study recruited 869 women living with HIV aged 45–60 years of pre‐, peri‐ and postmenopausal status across 21 HIV clinics in England from 2016 to 2017.

Follicle‐stimulating hormone was measured in a single serum sample from a subset of eligible participants and processed in local laboratories. Eligible women were those from 12 of the original clinical sites who reported irregular menses at the time of the main PRIME study visit and who, by the time of recruitment in this substudy (mean 2.7 years after the main visit) reported ≥ 12 months’ amenorrhoea. Women were excluded from participation if they had a history of hysterectomy and/or bilateral oophorectomy; reported current systemic hormone replacement therapy use (which affects serum FSH); had been pregnant or had breast‐fed in the past 12 months; or reported using hormonal contraception in the past 6 months for either contraceptive or non‐contraceptive reasons. Women received a £20 shopping voucher in recognition of their time.

The results of FSH testing were supplemented with data on menopausal symptoms [assessed using the Menopause Rating Scale (MRS) [15]] and lifestyle factors (smoking history, alcohol consumption and recreational drug use), collected through a self‐completed paper questionnaire. We obtained routine clinical data (BMI, current CD4 T‐cell count, current HIV RNA and current ART regimen) from clinical notes, and demographic information (such as year of birth and ethnicity) from participants' PRIME study data.

### Statistical methods

A previous study of FSH in postmenopausal women (without HIV) demonstrated that the 25th centile for FSH in this group was 30 mIU/mL [5]. Cejtin et al. [7], in their study of FSH levels in amenorrhoeic women living with HIV aged 16–55, reported a prevalence of elevated FSH of 47%. We therefore opted for 60% as a pragmatic estimate of prevalence of elevated FSH in this age group. Based on this, our planned sample size of 96 would have provided an estimate with 10% precision.

Serum FSH > 30 mIU/mL was defined as being consistent with postmenopausal status. This cut‐off is consistent with the standard cut‐off in most laboratory assays in the UK, and is in accordance with guidance from the Faculty of Sexual and Reproductive Healthcare stating that FSH > 30 mIU/mL is indicative of ovarian insufficiency [16]. Menopause symptom severity was measured by the MRS, a validated tool for assessing menopause‐related quality of life comprising 11 items in three symptom domains: psychological (e.g. depression and anxiety, irritability and fatigue), somatic (e.g. vasomotor symptoms and sleep disturbance) and urogenital (e.g. vaginal dryness, bladder symptoms and sexual problems) [15]. Each item is rated on a Likert scale between 0 (no symptoms) and 4 (severe symptoms). For this analysis, we used participants’ cumulative total MRS score to measure symptom severity, treating it as a continuous variable.

Sample characteristics were described using median/interquartile range (IQR) or proportions; associations between FSH and menopausal symptom severity were assessed using Pearson's correlations. All analyses were conducted in Stata Statistical Software: Release 16 (StataCorp LLC, College Station, TX, USA).

### Ethical approval

The baseline PRIME study had ethical approval from the South East Coast Surrey Research Ethics Committee on behalf of all NHS sites (REF 15/0735), and we had approval from the South Central Hampshire Research Ethics Committee (REF 18/SC/0570) for this substudy.

## RESULTS

Of 114 eligible women, 85 (74.6%) consented to participate in this study. Median (IQR) age was 53 (51–55) years; two‐thirds were Black African (*n* = 59); prevalence rates of current smoking (7.1%) and drug use (1.2%) were low and 15% (*n* = 13) reported high‐risk drinking (AUDIT‐C ≥ 5; Table 1). All were on ART, 76% (*n* = 65) had a CD4 T‐cell count > 500 cells/μL and 92% (*n* = 78) had an HIV viral load (VL) < 50 copies/mL.

**TABLE 1:** Characteristics of study sample

Variable	All [*n* (%)] (*N* = 85)	FSH > 30 mIU/mL [*n* (%)] (*N* = 81)	FSH ≤ 30 mIU/mL [*n* (%)] *(N* = 4)
Age (years) [median (IQR)]	53 (51–55)	53 (51–55)	52 (50–55)
Ethnicity			
Black African	59 (73)	55 (71)	4 (100)
White UK	4 (5)	4 (5)	0
Other^a^	18 (22)	18 (23)	0
Current smoker	6 (7)	6 (7)	0
Recreational drug use^b^	1 (1)	1 (1)	0
High‐risk drinking^c^	13 (15)	12 (15)	1 (25)
Nadir CD4 T‐cell count (cells/μL)			
> 500	1 (2)	1 (1)	0
≤ 500	59 (98)	55 (98)	4 (100)
Last CD4+ T‐cell count (cells/μL)			
> 500	65 (76)	62 (77)	3 (75)
≤ 500	20 (4)	19 (23)	1 (25)
Last HIV viral load < 50 copies/mL	78 (92)	75 (93)	3 (75)
Body mass index (kg/m^2^)			
< 25	12 (15)	12 (16)	0
25–29.9	22 (28)	22 (29)	0
30–34.9	25 (32)	25 (33)	0
≥ 35	20 (25)	16 (21)	4 (100)
Severe menopausal symptoms^d^	22 (28)	20 (27)	2 (50)

Abbreviations: FSH, follicle‐stimulating hormone; IQR, interquartile range.

^a^ Asian, mixed or other (but not specified).

^b^ Within last 3 months.

^c^ AUDIT‐C ≥ 5.

^d^ Menopause Rating Scale ≥ 17.

Overall, the median (IQR) FSH was 65.9 (49.1–78.6) mIU/mL. Only four women (4.7%; 95% confidence interval: 1.2–11.6) had FSH ≤ 30 mIU/mL (premenopausal levels); the median (IQR) FSH in this group was 19.2 (10.4–26.6) mIU/mL. Two of the four women had severe menopausal symptoms (MRS score ≥ 17); only one reported no symptoms. All four women with FSH ≤ 30 mIU/mL were Black African, none reported smoking or drug use, nadir CD4 T‐cell count was ≥ 200 cells/μL in two, most recent CD4 T‐cell count was ≥ 200 cells/μL in all, and only one woman had a detectable HIV viral load. The median BMI in these women was elevated compared with women with FSH > 30 mIU/mL (40.8 vs. 30.5 kg/m^2^); all four had a BMI ≥ 35 kg/m^2^.

**FIGURE 1:**
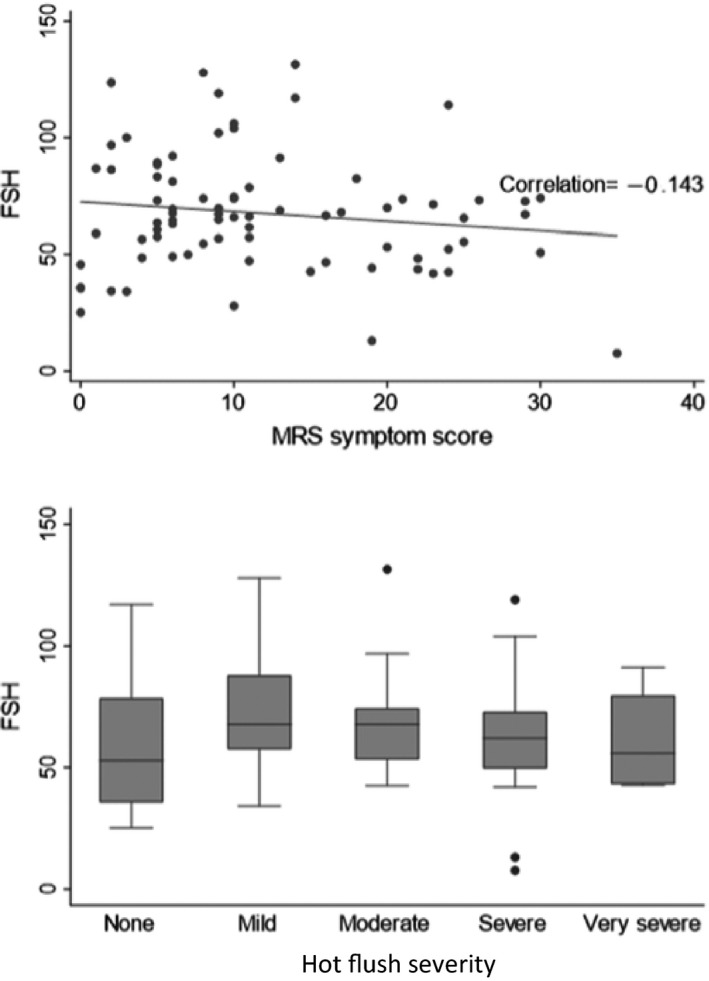
(a) Correlation between serum follicle‐stimulating hormone (FSH) levels and menopause symptom severity (total menopause rating scale (MRS) score); (b) median, interquartile range (box) and range (whiskers) FSH by hot flush severity (Menopause Rating Scale, MRS) (b)

The median (IQR) MRS in all women was 10.0 (5.0–19.0), and a quarter (22/78) reported severe menopausal symptoms (MRS score ≥ 17; Table 1). Symptoms in the somatic and urogenital domains were reported by 95.3% (81/85) and 67.1% (55/82) of women, respectively; all of those who completed the relevant section of the MRS (*n* = 83) reported psychological symptoms. We found no correlation between serum FSH and severity of menopausal symptoms overall (*r* = −0.14, *p* = 0.21; Figure 1a), or hot flush symptom severity specifically (median FSH no hot flushes, 52.8 mIU/mL; mild, 67.7 mIU/mL; moderate, 67.7 mIU/mL; severe, 62.0 mIU/mL; very severe, 55.9 mIU/mL; *p* = 0.37, Figure 1b).

## DISCUSSION

In this sample of 85 women living with HIV aged > 45 years and reporting ≥ 12 months’ amenorrhoea, 95.3% had an FSH > 30 mIU/mL, a level consistent with being postmenopausal. This suggests that ovarian decline due to menopause is the most likely cause of secondary amenorrhoea in women living with HIV aged > 45 years, and that FSH testing is not necessary for confirmation. Our findings differ from two previous studies reporting a high prevalence of amenorrhoea in women living with HIV without a rise in FSH [7, 8]. Both of these were conducted in the pre‐combination ART era; participants were predominantly ≤ 45 years, and a significant proportion had low CD4 T‐cell counts, with many reporting substance use (both of which are associated with secondary amenorrhoea). These studies are therefore unlikely to represent the contemporary population of women living with HIV in the UK aged > 45 years.

Four women in this study had a low FSH (≤ 30 mIU/mL), despite reporting ≥ 12 months’ amenorrhoea; all four had BMI ≥ 35 kg/m^2^. Our sample size is small, precluding us from drawing firm conclusions; however, this finding is consistent with the literature describing an attenuated FSH rise in menopause among women with high BMI [17, 18]. We suggest that clinicians be mindful of this association when interpreting FSH levels in potentially postmenopausal women living with HIV with high BMI. However, we also recognize that FSH levels may fluctuate and that the cross‐sectional nature of this study does not capture variations over time. In addition, we cannot exclude secondary amenorrhoea in these four women; obesity itself can result in changes in levels of gonadal steroid hormones, resulting in anovulatory cycles and irregular or absent menses [19].

We did not find a correlation between serum FSH and severity of menopausal symptoms (and specifically severity of hot flushes). Findings from other studies in women without HIV are inconsistent, although serum FSH has previously been reported to be associated with severity of vasomotor, musculoskeletal and mood symptoms [20, 21], pointing to a potential effect of FSH on menopausal symptoms independent of oestrogen depletion.

Our study is limited by small numbers. We did not have sufficient power to investigate differences between those with and those without elevated serum FSH, or to examine the correlation between FSH and severity of menopausal symptoms. As a cross‐sectional study, we lack longitudinal data on FSH which would allow us to describe and compare reproductive hormone trajectories over the menopause transition. Furthermore, we were unable to exclude the possibility of fluctuations in FSH in the four women with low FSH levels. Our study population is broadly representative of women living with HIV in the UK, with low prevalence of smoking and recreational drug use, both of which have been shown to be associated with secondary amenorrhoea in HIV. Therefore, our findings may not be generalizable to other settings where prevalence of these behavioural risk factors is higher. Finally, all study participants were on ART and we are therefore unable to extrapolate these findings to women not on ART.

However, this is the first study in the contemporary ART era to describe serum FSH in women living with HIV aged > 45 reporting ≥ 12 months’ amenorrhoea. Our findings indicate that postmenopausal status can be ascertained from menstrual history alone (without biomarker confirmation) in women living with HIV aged > 45 years who are on ART, as it is in women without HIV. Prior to the implementation of the NICE guidelines in 2015, it is estimated that 70% of FSH tests in England were requested on women aged > 45 years [22]. The consequent reduction in unnecessary FSH testing was predicted to save £9.6 million. Reducing unnecessary FSH testing in women living with HIV is therefore likely to lead to cost savings within already constrained services.

Most importantly, we hope our findings will allow clinicians to feel confident in assessing menopausal status in this patient group, without the need for further testing. This will improve recognition and management of menopausal symptoms in this population, therefore optimizing the health and well‐being of women living with HIV as they reach their post‐reproductive years.

## CONFLICT OF INTEREST

ST has previously received a travel bursary funded by Janssen‐Cilag through the British HIV Association, and speaker honoraria and funding for preparation of educational materials from Gilead Sciences. CS has received funding for membership in Data Safety and Monitoring Boards, Advisory Boards, speaker panels and for preparation of educational materials from Gilead Sciences, ViiV Healthcare and Janssen‐Cilag. FMB has received consultancy fees and conference support from Gilead Sciences. YG has received funding for Advisory Boards, preparation of educational materials and conference support from ViiV, Janssen‐Cilag and Gilead Sciences. NM has received honoraria for participation in advisory boards and educational webinars from ViiV, MSD and Gilead Sciences. IR has had conference support and consultancy fees from ViiV. RM has received speaker honoraria from Gilead.

## AUTHOR CONTRIBUTIONS

ST conceived and designed both the main PRIME Study and this substudy with support from FMB, RG and CS. RM was the Principal Investigator, supported by AS. HO, CS and ST conducted analyses. ST drafted the first version of this article. RM, HO and CS critically reviewed the first version of the article and all authors commented on and approved the final draft for publication.
